# A Case of Concurrent Mpox and Chlamydial Proctitis in a Patient With HIV

**DOI:** 10.7759/cureus.50918

**Published:** 2023-12-21

**Authors:** Rand Ibrahim, Alexander Pressman, Kimani Hicks, Carolyn G Brooks, Chiara Bettale, Jessica Nave, Hernan Bello, Daniel Dressler

**Affiliations:** 1 Department of Medicine, Division of Internal Medicine, Emory University, Atlanta, USA; 2 Department of Medicine, Division of Internal Medicine, Emory University School of Medicine, Atlanta, USA; 3 Department of Radiology and Imaging Sciences, Emory University School of Medicine, Atlanta, USA; 4 Department of Medicine, Emory University School of Medicine, Atlanta, USA; 5 Department of Medicine, Division of Hospital Medicine, Emory University School of Medicine, Atlanta, USA

**Keywords:** sexually transmitted infection (sti), rectal bleeding, monkeypox proctitis, hiv, mpox

## Abstract

The monkeypox (mpox) outbreak that began in May 2022 spread globally with a wide range of presentations. Mpox proctitis has been recognized as one of the severe forms of the virus during this outbreak. We present the case of a 33-year-old male with well-controlled HIV engaging in receptive anal intercourse presented with profuse rectal bleeding, tenesmus, and anal pain in July 2022. His symptoms persisted despite treatment for his rectal chlamydia with doxycycline. Rectal imaging with computed tomography demonstrated impressive inflammation. Contrast-enhanced images highlighted rectal wall thickening and submucosal edema. Diffuse lymphadenopathy of the anorectal region was also clearly seen. He received symptomatic treatment with tecovirimat resulting in the resolution of his symptoms and complaints. Subsequent rectal imaging displayed improvement and decreased inflammation. A better understanding of various presentations, imaging characteristics, and management is necessary to curb further dissemination.

## Introduction

The monkeypox (mpox) virus, first identified as a human pathogen in 1970, has largely been restricted to the African continent prior to the past 2 years [1]. In 2022, an outbreak began in the United Kingdom (UK) and has since spread globally, becoming a growing public health concern necessitating a better understanding of its transmission and presentations [2].

Typical complaints have included fevers, myalgias, lymphadenopathy, and a centrifugal asynchronous rash [3]. However, less frequently observed presentations, sometimes associated with delayed diagnosis and treatment, have been reported during the current outbreak.

The prevalence of mpox has been observed to be elevated among men who have sex with men (MSM), distinguishing mpox from other sexually transmitted infections (STI), which also occur at a higher rate among MSM than the general population, may be challenging [4,5]. A number of case reports with varied presentations have been reported among MSM, but most have involved the genito-urinary tract [6].

Monkeypox proctitis, increasingly recognized as a severe form of mpox presentation within the current outbreak, is debilitating to the patient and costly to the healthcare system [6-11]. Awareness of this form of monkeypox is vital for timely symptomatic relief. We present the case of a 33-year-old male with well-controlled HIV who regularly engaged in receptive anal intercourse and presented with profuse rectal bleeding, tenesmus, and anal pain in July 2022.

This article was previously presented as a meeting abstract at the 2022 Southern Hospital Medicine Conference on October 12, 2022, at the Emory Department of Medicine Conference on September 9, 2022, and at the Society of Hospital Medicine Conference on March 26, 2023.

## Case presentation

A 33-year-old man with a history of human immunodeficiency virus (HIV, diagnosed in 2007) presented to our hospital in July 2022 complaining of profuse rectal bleeding, pain, and tenesmus. His symptoms occurred over five days with increasing frequency and quantity. Mucous and clots accompanied the bleeding but no incontinence.

These symptoms were preceded by a prodrome of subjective fever, myalgias, a productive cough, and a papular skin rash. The lesions were described as both itchy and painful and underwent asynchronous crusting without pustular discharge or ulceration.

Sexual practices include receptive anal intercourse with multiple partners without the use of barrier protection. The most recent sexual encounter was three weeks prior to presentation with someone who later tested positive for mpox. No travel history was reported in the month prior to his presentation. Pertinent history included rectal chlamydia infection, detected on routine screening for sexually transmitted infections (STIs), for which he was receiving doxycycline for the 10 days prior to presentation. His HIV was well-controlled on Biktarvy (bictegravir/emtricitabine/tenofovir alafenamide) with undetectable viral load and most recent CD4 count of 542 cells/mm^3^ measured two weeks prior to presentation. He first presented to the ED 5 days prior to admission with the same complaints where his hemoglobin was found to be 14.8 mg/dL. He received mesalamine and was discharged.

**Figure 1:**
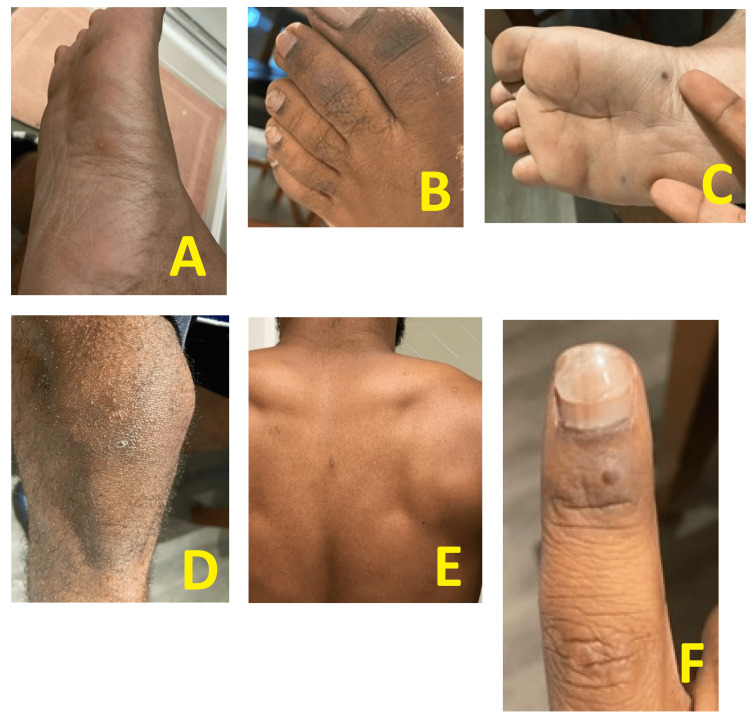
Maculopapular exanthem with isolated small pustules over along the palms and soles, back, and extremities Isolated small pustules on the feet (A-C), leg (D), back (E), and finger (F)

Upon presentation, he was found to be afebrile, with diffuse erythematous papular lesions, including the scrotum and base of the penis, but no perianal lesions. The papules were not groups, appeared at different time points, and had spread over the entire body (scalp, ears, nostrils, axilla, trunk, back, scrotum and penis, palms and soles, legs) (Figures 1A-1F).

**Figure 2:**
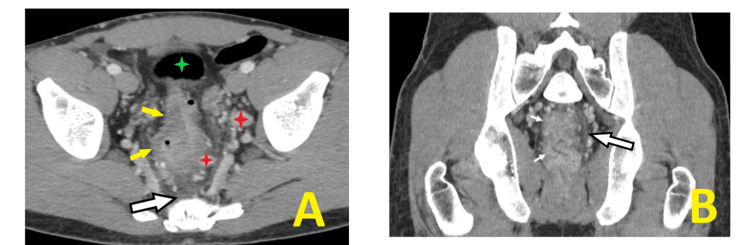
Contrast-enhanced pelvic CT demonstrating inflammatory proctitis A. Contrast-enhanced axial CT of the pelvis shows diffuse rectal wall thickening and submucosal edema (small yellow arrows), perirectal and presacral inflammatory fat stranding (larger white arrow with black border), and prominent mesorectal and left pelvic sidewall lymphadenopathy (red stars). Note the transition to a normal upstream bowel wall in the sigmoid colon (green star). B. Contrast-enhanced coronal CT of the pelvis shows similar findings of diffuse rectal wall thickening (small white arrows) and mesorectal inflammatory fat stranding (larger white arrow with black border).

His hemoglobin count was 12.5 mg/dL and remained above the transfusion threshold during his admission. On initial presentation, contrast-enhanced pelvic computed tomography (CT) demonstrated inflammatory proctitis with bulky adenopathy and diffuse rectal wall thickening with submucosal edema, perirectal and presacral inflammatory fat stranding, and prominent mesorectal and left pelvic sidewall lymphadenopathy (Figures 2A, 2b).

**Figure 3:**
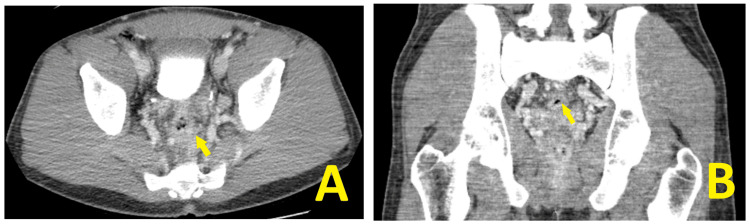
Contrast-enhanced axial and coronal CT of the pelvis A. Contrast-enhanced axial CT of the pelvis showing interval improvement/decrease inflammatory/infectious proctitis (yellow arrow). B. Contrast-enhanced coronal CT of the pelvis shows a similar decrease in proctitis (yellow arrow), without complete resolution of mesenteric and retroperitoneal lymphadenopathy.

Subsequent imaging, following therapy, demonstrated interval improvement in the inflammatory/infectious proctitis with decreased mesenteric and retroperitoneal lymphadenopathy (Figures 3a, 3b).

Swabs of the skin lesions were sent to the Department of Public Health for polymerase chain reaction (PCR) testing, confirming the mpox diagnosis. He received symptomatic management with laxatives for bowel movement and monitoring of hemoglobin levels during hospital admission. Following US Centers for Disease Control and Prevention (CDC) guidelines for his severe mpox presentation, he received antiviral therapy (tecovirimat, 14 days) alongside treatment for his rectal chlamydia and was instructed to self-isolate [12].

## Discussion

The recent mpox epidemic in the US has been disproportionately concentrated among individuals with high-risk sexual practices, notably MSM, suggesting high rates of viral inoculation into the anogenital region [2,5,8]. Abrasions of the rectal area during anal sexual intercourse can introduce the virus into the rectal walls, leading to a deeper inflammatory process.

Inflammation of the rectal walls, as seen on the CT scan of our patient, could spill over to profuse bleeding when extensive. More severe involvement of the rectal wall can also cause perforation. A delay in imaging and identification leads to a rectal wall perforation in a mpox proctitis case reported by Brown et al. [9]. Early imaging-contrasted, when possible, in patients with suspected mpox proctitis can facilitate appropriate patient triage and expedient management. Employing imaging can be paramount in the early characterization of viral and inflammatory involvement and subsequent disease control [9,10]. The use of imaging when suspecting deeper viral or internal inflammatory involvement is evolving. Discerning imaging indications and incorporating imaging findings into patient management will likely evolve as additional severe cases and case series are reported.

The coexistence of other “dormant” STIs in this high-risk population could also play a priming inflammatory role in leaving the gastrointestinal and genitourinary systems in a friable and susceptible condition, setting the stage for subsequent viral entry and bleeding. Català et al. reported that 76% of their cohort of patients in Spain were infected with coexisting STIs [7]. A high proportion of infected individuals (40% in the United States) have been noted to have a comorbid diagnosis of HIV [5]; it is thought that patients with concomitant HIV infection experience greater mpox disease severity [13]. It is, therefore, important to not abandon usual screening for other possible co-existing contributing STIs.

Maintaining a low threshold for suspecting mpox in the appropriate clinical and epidemiologic setting is important for timely intervention. In the current mpox outbreak, numerous cases have been observed of maculopapular lesions at ports of entry (e.g., skin, mucosal surfaces) followed by more invasive inflammatory manifestations, including in the pharynx and rectum or viscera (myocarditis) [11,12,14-17]. Management largely consists of symptomatic relief with isolation. Indications for tecovirimat (TPOXX®) therapy include severe disease (e.g., hemorrhagic disease, confluent lesions, sepsis, encephalitis, or hospitalization requirement), infections involving lesions in mucocutaneous sites, which may constitute a special hazard (e.g., eyes, mouth, genitals, or anus), and among special populations (e.g. immunocompromised patients, pregnant or breastfeeding individuals, pediatric patients, or patients with conditions compromising skin integrity) [11,12].

Curbing transmission through education, pre-exposure vaccination, timely identification, and isolation in communities at risk, while avoiding stigmatization, are necessary to control public health outbreaks of this type of communicable infection [5,18].

## Conclusions

The current mpox outbreak is presenting with a wide array of signs and symptoms not well-described in previous encounters with the virus. Maculopapular lesions at ports of entry (skin, mucosa) can be followed by more invasive inflammatory manifestations including the pharynx and rectum. A thorough elucidation of mpox transmission and the various forms of presentation are necessary for early detection, treatment, and isolation to prevent propagation. Thus far, the outbreak has been primarily reported in the MSM community, one that is already at high risk of STIs and confounding presentations. Efforts to understand and curb transmission (education of physicians and communities at risk, pre-exposure vaccination, timely identification, and isolation) in communities at risk, while avoiding stigmatization, are key to controlling this emerging public health concern.
